# Oral Cavity as a Source of Mesenchymal Stem Cells Useful for Regenerative Medicine in Dentistry

**DOI:** 10.3390/biomedicines9091085

**Published:** 2021-08-25

**Authors:** Ilaria Roato, Giorgia Chinigò, Tullio Genova, Luca Munaron, Federico Mussano

**Affiliations:** 1 Department of Surgical Sciences, CIR-Dental School, University of Torino, 10126 Torino, Italy; federico.mussano@unito.it; 2 Department of Life Sciences and Systems Biology, University of Torino, 10123 Torino, Italy; giorgia.chinigo@unito.it (G.C.); tullio.genova@unito.it (T.G.); luca.munaron@unito.it (L.M.)

**Keywords:** oral cavity, mesenchymal stem cell, periodontitis, dental pulp

## Abstract

The use of mesenchymal stem cells (MSCs) for regenerative purposes has become common in a large variety of diseases. In the dental and maxillofacial field, there are emerging clinical needs that could benefit from MSC-based therapeutic approaches. Even though MSCs can be isolated from different tissues, such as bone marrow, adipose tissue, etc., and are known for their multilineage differentiation, their different anatomical origin can affect the capability to differentiate into a specific tissue. For instance, MSCs isolated from the oral cavity might be more effective than adipose-derived stem cells (ASCs) for the treatment of dental defects. Indeed, in the oral cavity, there are different sources of MSCs that have been individually proposed as promising candidates for tissue engineering protocols. The therapeutic strategy based on MSCs can be direct, by using cells as components of the tissue to be regenerated, or indirect, aimed at delivering local growth factors, cytokines, and chemokines produced by the MSCs. Here, the authors outline the major sources of mesenchymal stem cells attainable from the oral cavity and discuss their possible usage in some of the most compelling therapeutic frontiers, such as periodontal disease and dental pulp regeneration.

## 1. Introduction

In the last decades, the advancement of biotechnologies has held the promise to disrupt the biomedical field with innovative protocols [1]. These ambitious goals, set in late 1990 with great enthusiasm, seem to have finally become implementable in the dental field, which may be on the verge of attaining important results. Here, the authors wish to recapitulate the most compelling updates dealing with dentistry.

**Figure 1:**
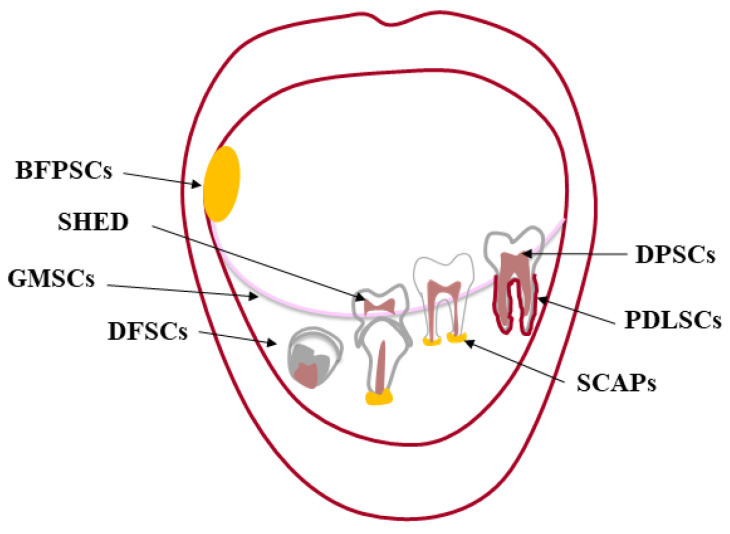
Oral cavity and its sources of dental MSCs. The oral (or buccal) cavity is the upper end of the alimentary canal in higher vertebrates bounded by “the lips anteriorly, the cheeks laterally, the palate superiorly, the floor of the mouth inferiorly, the oropharynx posteriorly”. The mandible and maxillae are the bony structures of the oral cavity to which the teeth articulate (gomphosis). Each tooth consists of two parts: one intraoral (the crown) and the other one endosseous (the root). The crown is made of enamel, mainly hydroxyapatite, and dentin forming the bulk of the tooth along with the dental pulp. This soft tissue providing blood supply and innervation is housed within the dentin from the tip of the root to the crown [20]. A layer of cementum covers the root, anchoring the tooth to its bony socket through the periodontal ligament. This sophisticated supporting structure along with the gingiva adjacent to the tooth is called periodontium. In the oral cavity, there are different sources of MSCs: dental pulp stem cells (DPSCs); pulp tissue of exfoliated deciduous teeth (SHED); periodontal ligament (PDLSCs); apical papilla of developing teeth (APSCs); dental follicle (DFSCs) and gingiva (GFSCs); buccal fat pad (BFPSCs).

The premise to any kind of tissue engineering approach, adult stem cells are undifferentiated cells, present in almost every tissue [2], that can both renew themselves, keeping their “stemness”, and differentiate into a variety of histotypes [3,4]. Stem cells were first found in the bone marrow (BM), which harbors hematopoietic stem cells (HSC) [5,6,7] and mesenchymal stem cells (MSCs) [8]. The oral cavity is a large source of MSCs, localized in specialized well-characterized tissues [9,10] (Figure 1). The first type of dental stem cells was isolated from the human pulp tissue of a third molar and termed “postnatal dental pulp stem cells” (DPSCs) [11,12]. Later, other types of dental MSCs have been described according to the different site of isolation: pulp tissue of exfoliated deciduous teeth (SHED) [13]; periodontal ligament (PDLSCs) [14]; apical papilla of developing teeth (APSCs) [15,16]; dental follicle (DFSCs) [17], gingiva (GFSCs) [18], and buccal fat pad (BFPSCs) [19].

In this review, we reported and discussed the recent literature concerning oral MSCs, focusing on their potential application to treat two common pathologies associated with the oral cavity, periodontal disease and destroying caries, which seriously damage teeth and need the regeneration of the dental-pulp complex.

## 2. Oral MSCs

MSCs in the oral cavity are responsible for the maintenance and repair of their associated tissues [21]. Even though dental MSCs show features shared with BM-MSCs as initially reported by Pittenger et al. [6,22], they differ in neurogenic potential due to their origin from the neural crest during embryonic development; indeed, the dental mesenchymal tissue is also called “ectomesenchyme” for its interaction with the neural crest [23]. Moreover, dental MSCs are more committed to odontogenic than to osteogenic development [24], since MSCs derived from specific tissues retain some “memory” of those tissues and, thus, exhibit some tissue-specific properties in addition to more generic multipotential, and these can be defined by their niche environment [24,25]. According to the International Society for Cellular Therapy, dental MSCs show plastic adherence ability; they are positive for CD90, CD105, CD73, and CD44 and negative for hematopoietic markers CD34, CD38, CD45, and CD54. Dental MSCs are able to differentiate into osteoblasts, chondroblasts, and adipocytes [26].

### 2.1. Dental Pulp Stem Cells (DPSCs)

These cells exhibit the canonical MSCs properties, such as multi-lineage differentiation capabilities, high proliferation rate, and immunomodulatory activity [12,26]. Moreover, DPSCs have neurogenic potential due to their origin from the neural crest [27]. As they reside in a perivascular niche in the postnatal dental pulp tissue [28], likely deriving from pericytes [29], they can contribute to angiogenesis in vivo [30]. DPSCs’ ability to differentiate into endothelial cells and their angiogenic potential is also due to the production of vascular endothelial growth factor (VEGF). DPSCs have been used to regenerate a vascularized dentin-pulp-like complex in empty root canal spaces [31]. In a pilot clinical study, DPSCs pre-treated with G-CSF were implanted in the empty root canal of traumatized permanent incisors of five patients with irreversible pulpitis, observing a vascularized and nervous reconstruction of pulp tissue [32]. In 2018, Xuan et al. reported the results of a randomized clinical trial in which teeth with necrotic pulps were transplanted with DPSCs’ aggregates in situ. A successful three-dimensional regeneration of the whole dental pulp tissues occurred, including an odontoblast layer, connective tissues, blood vessels, and even neuronal markers [33].

DPSCs have the peculiar potential to differentiate into odontoblasts able to repair dentin [34]. In vivo ectopic transplantation of DPSCs, mixed with hydroxyapatite/tricalcium phosphate formed a dentin-pulp-like complex associated with vascularized pulp-like tissue [35]. In one study comparing donor matched BMMSCs and DPSCs, alkaline phosphatase activity was significantly higher in DPSCs than in BMMSCs after three weeks of induction in osteogenic medium [36]. DPSCs showed mineralization potential [12,36,37,38]; indeed, bone formation by human DPSCs has been shown both in vitro and in vivo [39,40,41,42,43]. The potential of DPSCs for periodontal regeneration may be questionable because of their limited capacity to form cementum [43,44]. A recent revision of the literature reported that DPSCs on synthetic scaffolds are useful to treat bone defects, showing encouraging results of early new bone formation in preclinical animal studies [45].

### 2.2. Stem Cells from Exfoliated Deciduous Teeth (SHED)

Similar to DPSCs, SHED derive from dental pulp. However, due to the developmental differences between deciduous and permanent teeth, SHED express higher levels of genes related to stemness (OCT4, SOX2, NANOG, and REX-1) compared to DPSCs, retaining a higher plasticity through passaging in vitro [45,46]. SHED are highly proliferative and capable of differentiating into a variety of cell types, such as neural cells, osteoblasts, chondrocytes, and adipocytes [13].

SHED can differentiate into odontoblasts even though they may show lower reparative efficacy than the odontoblasts derived from DPSCs [47]; indeed, they form dentin-like or pulp-like tissue but not the dentin-pulp complex [13]. Only when combined with collagen I and injected into full-length human root canals do SHED form the dentin-pulp complex, thus, this can be a strategy to facilitate the completion of root formation in necrotic immature permanent teeth [48].

SHED may also have perivascular origins with pericyte-like characteristics, they can differentiate into endothelium [49] promoting vascularization. In vivo studies revealed that SHED are able to form functional vessel-like structures upon transplantation [50]. We also reported that SHED, maintained in osteogenic conditions, significantly increase the pro-angiogenic signature [51]. More recently, Kondo et al. confirmed the pro-angiogenic effect of SHED, which secrete pro-angiogenic factors for primary endothelial cells [52].

The osteoinductive potential of SHED has been investigated in vivo: SHED repair critical size calvarial defects with effective bone formation [53]. To improve the osteogenic potential of SHED, they have been cultured in chitosan scaffolds containing divalent metal phosphates, showing a significant increase in osteoblastic differentiation compared with cells cultured without divalent metal phosphates [53,54].

### 2.3. Periodontal Ligament Stem Cells (PDLSCs)

Human PDL contains a group of stem cells (PDLSCs) that express MSCs’ surface markers, present self-renewal ability, and have multipotent capacity [55], being able to differentiate into cementoblasts/osteoblasts, adipocytes, and collagen-forming cells [14]. Thus, PDLSCs are responsible for regenerating and maintaining periodontal tissue homeostasis, tooth-bone attachment, and masticatory function. PDLSCs are the most studied and considered the most suitable source for periodontal regeneration; they are easily accessible and capable to secrete mineralized structure.

The osteoinductive potential of PDSCs is less prominent than for DPSCs and SHED [56,57,58], but they can regenerate PDL tissue [58], because in vivo, they are able to differentiate into cementoblasts and to form collagen fibers embedded in cementum-like tissue. The presence of the TGF-β1 signaling basically determines whether hPDLCs are differentiated into ligament progenitors or cementoblasts. Indeed, the inhibition of TGF-β1 blocks cementoblastic and promotes fibroblastic differentiation of the ligament progenitors [59]. Indeed, in a rat model, typical PDL-like structures were generated after PDLSCs transplantation in a periodontal lesion, where PDLSCs generated PDL attachment in vivo by forming Sharpey’s fiber-like collagen bundles that were connected to cementum-like structure [14]. Moreover, PDLSCs express scleraxis, a tendon/ligament-specific transcription factor, at higher level compared to BMMSCs or DPSCs, suggesting PDLSCs enhanced ability to regenerate PDL tissue [14].

PDLSCs carried by hydroxyapatite/tricalciumphosphate (HA/TCP) have the potential to form cementum/PDL-like structure in vivo [15]. In the last years, a strong interest concerns also the secretome of PDLSCs; indeed, transplantation of PDLSC-conditioned medium (CM) has been investigated for its power to induce new PDL attachment and bone defect regeneration in rat models of periodontal defects. According to Nagatai et al., more recently, a compound of concentrated growth factor and PDLSCs-CM resulted effective in promoting cell proliferation of PDLSCs, proving this product useful for future applications in periodontal tissue regeneration [60].

### 2.4. Stem Cells from the Apical Papilla (SCAPs)

Apical papilla is the soft tissue at the apices of developing permanent teeth; it is the precursor tissue of radicular pulp, enriched of stem cells with highly proliferative potential. SCAPs are easily obtained from the soft tissue loosely attached to the apices of immature permanent teeth, such as the third molar [16]. The dental papilla is the tissue responsible for the formation of the dentin-pulp complex, thus, SCAPs have been studied for their regenerative potential [61].

SCAPs display a greater potentiality to remodel dentin than DPSCs [15], and they can differentiate into dentin on the surface of HA/TCP scaffolds [16]. SCAPs are involved in root development and regeneration. In minipigs, SCAPs and PDLSCs were transplanted, inducing root and PDL tissue renewing [62]. Reconstruction of complex critical-size defects (CSD) in the craniofacial region is challenging and exosomes derived from SCAP (SCAP-Exo) promote tissue regeneration of palatal gingival CSD in vivo by increasing vascularization. Indeed, the migration of endothelial cells was enhanced by improving their cytoskeletal reorganization [63].

### 2.5. Dental Follicle Stem Cells (DFSCs)

DFSCs reside in the connective tissue loosely surrounding the developing tissue; they are responsible for the formation of alveolar bone and the root-bone interface. Their retrieval is linked to tooth extraction [64]. Compared to the other dental MSCs, DFSCs show a higher proliferative potential and osteogenic properties [64,65,66]. DFSCs are more immature and express more DSPP than PDLSCs. Indeed, they show a marked odontogenic potential [67], being able to regenerate dentin and have potential capabilities of periodontal differentiation and root regeneration. DFSCs can form PDL-like structures in vitro [17]. Upon in vivo transplantation, DFSCs can renew root by producing cementum-like tissue and PDL collagen fibers [65]. DFSCs express higher levels of osteogenic markers such as RUNX2 and ALP than DPSCs and SHED [66]. Recently, isolated dental follicle epithelial stem cells from DFSCs were also found to form salivary gland cells and ductal cells. DFSCs play also an active role in the treatment of inflammatory diseases and autoimmune diseases in animal models [68].

### 2.6. Gingival Mesenchymal Stem Cells (GMSCs)

GMSCs were isolated and characterized by Mitrano et al.; they satisfy the minimal requirements for MSCs, showing multilineage differentiation abilities, expressing MSCs markers, and growing in adherence [18]. Different from other dental MSCs, GMSCs show high accessibility and do not need tooth extraction for their harvesting. Indeed, GMSCs are easily accessible from healthy or inflamed gingiva and are readily found in discarded dental tissue samples [69]. GMSCs showed immunomodulatory capacity as the other dental MSCs; indeed, they induce an anti-inflammatory macrophage polarization and inhibit osteoclast, reducing periodontal bone resorption in a mice model [70].

GMSCs osteogenic potential was demonstrated in vitro but not in vivo [68], even though recently EVs derived from GMSCs expressed a high level of RUNX2 and BMPs and promote extracellular matrix and mineralized nodules of new bone [71]. Upon transplantation into gingiva lesions of rats, GMSCs regenerated normal tissue [72]. CM from GMSCs showed a similar ability to the one from PDLSCs to induce periodontal ligament regeneration in rats [48].

### 2.7. Buccal Fat Pad Stem Cells (BFPSCs)

Recently, buccal fat pad usually called Bichat’s fat pad, emerged as source of stem cells (BFPSCs), which resulted successful to repair bone defects of the jaws, alone [73] or in combination with inorganic bovine bone mineral [74]. BFPSCs were comparable to DPSCs in terms of osteo-differentiating ability, thus they can be used for bone regeneration protocols [75].

## 3. MSCs-Based Therapeutic Approaches

The use of MSCs for regenerative purposes and, in particular, for bone regeneration represents a challenge, thus, many studies have been conducted to investigate the osteogenic potential of MSCs derived from different sites. The regenerative capabilities of MSCs derived from different regions of the oral cavity have been shown in Table 1, whereas their osteogenic potential has been deeply investigated, and readers interested in this field are referred to dedicated reviews on the matter [76]. Dentistry may benefit from innovative protocols entailing MSCs. Among the most relevant diseases, in terms of prevalence or health burden, one must consider periodontal disease and destroying caries, demanding, respectively, the regeneration of the periodontal and dental-pulp complex.

### 3.1. Periodontal Diseases

As the sixth most prevalent disease in the world, periodontal diseases (PDs) are chronic inflammatory conditions affecting the periodontium, triggered by the microbial biofilm of dental plaque [78], which contains up to 800 different species [79]. Although putative pathogens include a variety of microorganisms ranging from Gram-negative anaerobic bacteria to spirochetes, encompassing even viruses, no single pathogen is likely to cause autonomously the disease; rather it is due to the imbalance of the microbial biofilm [80]. Beginning with the localized inflammation of the gingiva (gingivitis), PD may progress, if untreated, to chronic periodontitis, which is characterized by deep periodontal “pockets”, a hallmark of the disease, due to the destruction of tooth-supporting tissues [79,80,81,82]. Epithelial cells prevent microorganisms from reaching the periodontal ligament through their sealing junction in a healthy subject, but they are also the sentinels that elicit an immune response owing to their resident dendritic Langerhans cells. The latter presents the microbial antigenic material to the lymphocytes, thus, triggering the infiltration of neutrophils, granulocytes, and lymphocytes into the periodontal lesion [83]. The consequent severe chronic inflammatory response sustained by the osteoclasts is responsible for the formation of granulation tissue [84]. Upon reaching the site of damage, B cells become plasma cells, whose antibodies are important in modulating the onset of periodontitis. The role of T cells, particularly that of CD4+ T helper cells in this pathology, has been deeply investigated, with some contradictory results, likely because different T-cell subsets predominate at distinct phases of the disease [85]. More recently, the role of Th17 and its key cytokine IL-17 in the pathogenesis of periodontitis has been investigated, as revised by Bunde et al. [86].

Periodontal therapy is theoretically aimed both at stopping the disease progression and at regenerating the periodontium. The former task proved easier to be attained than the latter, which has remained a clinical challenge [82]. Researchers have envisaged the use of MSCs to treat periodontal defects with two main approaches: (a) exploiting the immunomodulatory potential of MSCs and (b) renewing the bone–ligament–cementum complex through tissue engineering protocols.

#### 3.1.1. Exploiting the Immunomodulatory Potential of MSCs

In periodontitis, the rate of inflammation correlates with the severity of the disease [87]. PDLSCs derived from healthy periodontium protect tissue from ROS-mediated damages by suppressing the production of ROS by neutrophils [86,87,88,89]. Oral MSCs interact with the innate and adaptive immune system; indeed, they escape immune recognition and exert anti-inflammatory and immune-modulatory effects via the suppression of T-, B-, natural killer, and dendritic cells, both in vitro and in vivo [90].

For instance, DPSCs and GMSCs can interfere with the maturation and activation of dendritic cells, reducing their antigen-presenting cell ability. They also promote the anti-inflammatory phenotype of macrophages, increasing prostaglandin-E2 (PGE2), IL-6, and IL-10 [91]. DPSCs inhibit proinflammatory macrophages modulating the TNF-α/IDO axis [92]. A dysregulation of T cells associated with inflammatory conditions concerns the balance between Th17 and T reg; DPSCs, SHED, PDLSCs, and GMSCs suppress Th17 cells and promote Treg, reducing the inflammation [86,93]. Moreover, oral MSCs inhibit peripheral blood mononuclear cell proliferation through secretion of indoleamine 2,3-dioxygenase (IDO), transforming growth factor-b (TGF-β), and hepatocyte growth factor (HGF) [69,92,93]. DPSCs and GMSCs abolish the proliferation of NK cells and Th1 by activating the Fas/Fasl pathway [94]. PDLSC and DPSCs also show an inhibitory effect on B cell proliferation, differentiation, and antibody production [95].

**Figure 2:**
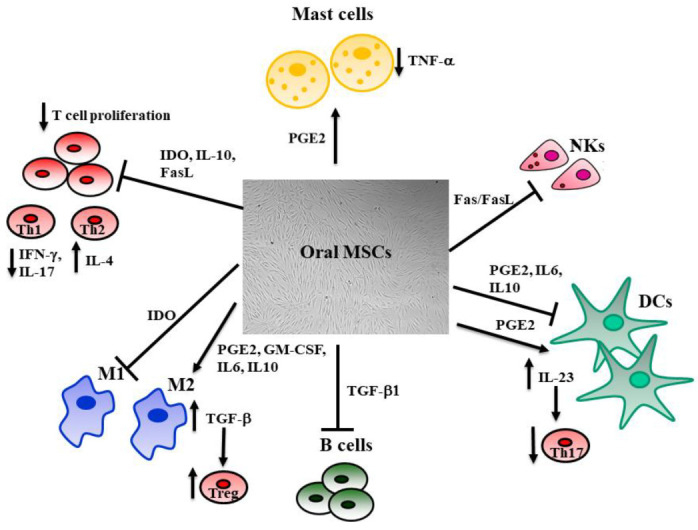
The influence of MSCs on immune system cells. MSCs regulate local inflammation by interacting with innate and adaptive immune system cells. The antigen-presenting cell activity mediated by dendritic cells (DCs) is downregulated by MSCs through the inhibitory effect due to IL-10, IL-6, and PGE-2. This last also increases the release of IL-23, which stimulates Th17. MSCs inhibit the pro-inflammatory M1 macrophages through the activation of the indoleamine 2,3-dioxygenase (IDO) pathway and promote M2 macrophages, increasing the release of PGE-2, IL-6, IL-10, and GM-CSF. M2 also activates T-regs through the TGF-β pathway. T and natural killer (NK) cell proliferation is inhibited by MSCs’ release of IL-10, IDO, and activation of the Fas/Fas Ligand pathway, resulting in reduced production of IFNγ and IL-17 by T helper 1 (Th1), whereas Th2 enhance their production of IL-4. The release of TGF-β1 hinders B cell proliferation. MSCs inhibit mast cells’ release of TNF-α through the activation of the PGE-2 axis. This regulation of the immune system exerted by MSCs is fundamental, since silencing the immune response during tissue repair is necessary to its remodeling.

The ability to isolate and expand MSCs in vitro without losing their phenotype or multilineage potential allows their use for tissue repair [96]. The administration of MSCs results in several effects, such as differentiation, secretion of numerous cytokines and growth factors, immune-modulation, and angiogenesis, which are all thought to contribute to the regeneration of damaged human tissues. It is increasingly evident that MSCs transplantation results in a low engraftment rate. MSCs’ survival after injection in inflamed tissue is short, with a half-life of 24 h [97]. For instance, SHED injected in a periodontitis-induced defect diffused a little during 3 days after injection, then rapidly decreased [98]. This is consistent with the observation that BMMSCs injected in an injured cornea almost disappeared after 3 days, while inflammation and partial healing effects were detectable [99]. These results proved that a high number of injected MSCs is not causally linked to the observed effect [100], because MSC therapeutic effects seem to be related to the paracrine secretion of soluble mediators; secretomes, including cytokines; peptides; proteins; microRNA; metabolites; and extracellular vesicles, such as exosomes, which exert their activity by modulating the immune response. Indeed, the immune-modulatory properties of MSCs (Figure 2) depend on both direct cell-to-cell contact as well as by the release of different factors, such as IDO, nitric oxide (NO), TGF-β1, IL-1, IL-6, IL-10, and PGE2 [88,99]. MSCs can be considered both sensors and regulators of inflammation in a specific tissue; indeed, their action on the surrounding environment is strictly linked to the rate of inflammation [100,101]. The regulation of the immune system exerted by MSCs is relevant since silencing the immune response during tissue repair is necessary to induce tissue regeneration. Right after an injury, MSCs promote inflammation through soluble factors and cytokines release, which promotes the recruitment of immune cells to the local area, but when inflammatory cytokines exceed a certain threshold, MSCs can activate an anti-inflammatory response to allow tissue repair. MSCs inhibit effector T cells under high concentrations of IFNγ and TNFα, while with a low concentration, MSCs promote T cell proliferation [102,103,104].

Another approach of MSC-based cell therapy takes advantage of MSCs’ release of exosomes that exert biological effects on the local microenvironment or at distant sites. Growing evidence suggests that exosomes act as an important regulator in oral diseases, thus, the application of oral MSCs-derived exosomes might assume a crucial role as a therapeutic approach for tissue regeneration in different oral pathologies, such as ONJ, periodontal disease, and oral oncotherapy [103,104,105].

In periodontal disease, PDLSC-exosomes make a fundamental contribution to the maintenance of periodontal immune/inflammatory homeostasis. For instance, PDLSC-exosomes are responsible for the unbalance of Th17/Treg in periodontal tissue of patients with periodontitis. Compared with exosomes extracted from normal PDLSCs, exosomes derived from LPS-stimulated PDLSCs contain a higher amount of miR-155, which reduces Th17 but increases Treg, decreasing inflammation through the Th17/Treg/miR-155 regulatory network [106]. Moreover, PDLSC-exosomes are involved in the regulation of bone remodeling during periodontal inflammation [107]. A recent work reported an anti-inflammatory action of PDLSC-exosomes during the interaction between PDLSCs and macrophages [108].

Exosomes derived from SHED resulted more effectively in stimulating the osteogenic potential of PDLSCs since they activate Wnt/β-catenin and BMP signaling pathways [109]. SHED-exosomes regulated the anti-inflammatory immune response in a mouse model of acute lung injury [110].

EVs derived from GMSCs have anti-inflammatory potential through the production of a significant amount of interleukin 1 receptor antagonist, which acts as an antagonist against the proinflammatory cytokine IL-1 and downregulates TNFα to mediate inflammation [111]. GMSC-exosomes reduce oxidative-stress-induced cellular senescence, which is a condition able to stimulate inflammation and induce different pathologies, such as periodontitis [112]. GMSC-exosomes promote wound healing in diabetic mice by stimulating collagen remodeling, angiogenesis, and re-epithelialization [113].

There is a growing interest in the role played by the inflammatory/immune response in the pathogenesis of periodontitis [114]. Du et al. have proposed the direct injection of allogeneic bone-marrow-derived MSCs into the periodontal defect of rats, suggesting how powerful the anti-inflammatory and immunomodulatory function may be in the periodontal repair [115]. However valuable, in vivo models only give proof-of-concept preparatory evidence that is to be construed as preliminary to human randomized clinical trials and systematic reviews. No such level of evidence has been achieved so far, unfortunately, in this field.

#### 3.1.2. Regenerating the Periodontium

Among the currently available procedures, guided bone regeneration entails the placement of a membrane barrier under the soft tissue (to reduce the risk of infection) as a scaffold or as a holding device for bone or bone substitute grafts [116]. Membranes delivering antimicrobial or growth-stimulating agents are also available [117]. From an anecdotal point of view, the case report of a bone defect treated with a 3D-printed polycaprolactone-based scaffold, enriched with platelet-derived growth factors, which was still in place at one-year follow-up, is noteworthy [118]. However, skepticism remains considering that, often, implanting bone substitute materials into the periodontal defects resulted in the long junctional epithelium, rather than in well-organized fibers connecting the adjacent cementum and bone [119].

For an ideal PDL regeneration to occur, highly organized collagen fibers should be properly re-inserted perpendicularly to bone and cementum. The importance of using MSCs for regenerating the periodontium was demonstrated with a dog model [120], which could highlight the active role of these cells in outperforming the natural repair. Several pre-clinical studies have described the formation of new PDL-like tissues via the delivery of PDLSCs [121] BMMSCs [115], ASCs [122], and even induced pluripotent stem cells (iPSCs) [123].

To date, according to Bartold et al., “the large number of animal studies carried out have clearly shown that PDLSCs have the potential to form bone, cementum, periodontal ligament-like structures and enhance overall periodontal regeneration” [117]. In comparison to other MSCs, PDLSCs seem more suitable for periodontal tissue engineering [122,123]. The consideration that very few cells attach to the recipient surfaces (alveolar bone and cementum) has prompted the implementation of cell sheet technology.

First described in 1993 [124], this technique is based on the use of poly-N-isopropyl acrylamide (PIPA Am), as a cell culturing substrate. PIPA Am can support the growth of cell monolayers at 37 °C and, owing to a temperature-sensitive unique feature, release them at temperatures below 20 °C, avoiding the usual enzymatic degradation of integrins through trypsin-EDTA. This is essential to maintain the integrin–fibronectin complex, whereby, possibly enhancing the adhesion of the cell sheet to the denuded root surface [125]. Several in vivo studies support this therapeutic approach [124,125,126,127] and made it conceivable to run a human trial [117].

Despite the abundance of preclinical studies [128], the clinical effectiveness of periodontal tissue engineering based on PDLSCs seems far from achieved. Stem cell therapies are clinically in their infancy and may be hindered by safety and regulatory issues. The long-term success of innovative procedures needs to be assessed in well-designed RCTs before becoming standard of care. According to Novello et al. [129], up to 2019, only two small RCTs using, respectively, PDLSCs [130] and DPSCs [131] could be included in a meta-analysis with inconclusive evidence, suggesting a limited impact of MSC-based therapy on periodontal regeneration. High-quality RCTs are needed to determine the efficacy and safety potential of MSCs in this context.

### 3.2. Dental Pulp Restoring

Enamel and dentin are dissolved by acid-forming microorganisms during caries formation [132]. In proximity to the pulp, dentin contains odontoblast processes that are capable of perceiving external stimuli [131,132]. Intratubular deposition of minerals is the first response to pathologic stimuli, followed by the formation of reactionary dentin [133,134]. Rapidly progressing caries, however, may imbalance this mechanism by disrupting the odontoblast layer, thereby, recruiting and activating MSCs to form reparative dentin (tertiary dentin) to the site of action [20]. Moreover, as soon as microorganisms reach dentin, an infection-related immune response is elicited within the pulp [135], spanning from an initially reversible, local inflammation to irreversible pulpitis [136]. Odontoblasts, supported by dendritic cells, trigger the innate immune response through antigen presentation [137]. Upon activation of toll-like receptors (TLRs), a type of pattern recognition receptors (PRRs), proinflammatory cytokines are produced, recruiting circulating immune cells [138]. As the caries lesion deepens, the immune response intensifies, leading to the accumulation of lymphocytes, neutrophilic granulocytes, and macrophages [136]. In addition, small blood vessels start sprouting in the injured area of the pulp, while dendritic cells interact with nerve fibers [139].

Therefore, to prevent pulp degeneration, it is mandatory to limit the inflammatory and immune reaction, which can only be achieved if the microbiological insult is timely kept under control through the removal of caries and cavity sealing. An intact odontoblast layer enables proper healing, while loss of the odontoblast layer, owing to pathologic noxae or pulp exposure, entails their replacement by odontoblast-like cells. These cuboidal cells secrete reparative dentin, subverting the normal histology of the dentin–pulp interface. Provided that the pulp has not undergone irreversible inflammation, it is possible to apply bioactive materials to the exposed pulp (direct pulp capping) to facilitate the deposition of reparative dentin, the so-called bridging [140]. To improve this option, clever attempts have been made to design advanced materials such as biodegradable collagen sponges imbued with low doses of small molecule glycogen synthase kinase (GSK-3) antagonists. In their study on mice, Neves et al. [141] reported scaffold colonization by pulp cells resulting in almost complete mineralization and closure of the lesion. The same model was applied to rats, suggesting that this enhancement of natural reparative dentinogenesis holds potential for future clinical applications [142]. Although this approach has proven unable to attain tubular dentin, it paves the way toward sophisticated repair strategies.

As the inflammatory process of the dental pulp is localized, affected areas may be selectively amputated (partial or total pulpotomy) in the course of treatment [134,142,143], possibly promoting healing of the remaining tissue [144]. If the microbiological load is so strong as to induce an uncontrolled thorough inflammation, the pulp undergoes necrosis, and root canal therapy is required, which implies disinfection and filling of the root canals with a synthetic material. This may hinder the long-term survival of teeth whose root development has not been completed [145]. Pulp regeneration could enable root development, ensuring better outcomes [146]. This goal was first attempted in the early 2000s [146,147] by inducing canal bleeding and adding bioactive cement. Although histologically different from the pulp, a vascularized and innervated tissue was obtained [148,149], capable of maintaining thermal sensitivity and ensuring root completion [150,151,152]. This procedure, known as “guided endodontic repair” [153], is currently performed following the guidelines and indications issued by professional societies [154].

Researchers have, nevertheless, focused their attempts on more ambitious strategies, possibly resulting in regeneration rather than repair. Several concepts have been proposed and assessed in terms of clinical feasibility over the past decade [155]. Stem cell therapy with autologous CD105+ cells was proposed successfully in dogs [156]. Briefly, after pulpectomy in fully formed teeth, ex vivo expanded progenitor cells were loaded on carriers with stromal cell-derived factor-1 (SDF-1) and transplanted into root canals. By day 14, a complete restitution ad integrum occurred, including nerves and blood vessels, which is consistent with the expression of angiogenic/neurotrophic factors described in CD105+ cells [157]. This approach was also proven in human patients who had autologous mobilized DPSCs reinserted into pulpectomized teeth through atelocollagen scaffolds with granulocyte colony-stimulating factor (G-CSF) [31]. Similarly, promising results could be attained in injured immature permanent teeth by using SHED in a controlled clinical trial [33].

Albeit fascinating, the procedure described above seems hardly transferrable to everyday clinical practice. To harvest cells, one needs at least either a healthy tooth to be discarded or previous storage in a cell bank [158]. Moreover, cell isolation and expansion are time-consuming, expensive, and usually not performed by clinics.

Therefore, over the last years, in situ tissue engineering has gained increased interest. It aims to exploit endogenous stem cell sources [159] to bypass ex vivo cell manipulation. To harness inherent bodily regeneration, it is paramount to prepare a suitable milieu that enhances the homing of local cells [160]. After cell colonization, capillaries and nerve fibers may grow within the scaffold, eventually mimicking the original pulp. A key factor is the selection of the best signaling cues endowed with chemotactic and proliferative activity, namely, growth factors, and a variety of proteins [158,160,161]. Furthermore, many studies have been focused on assessing the optimal material features to develop customized scaffolds for pulp tissue engineering [162,163,164,165,166]. Should part of the dental pulp be intact and vital, cells could be conveniently mobilized from it into a scaffold material [167].

MSC-derived exosomes can be useful also for dental pulp regeneration, leading to the increased expression of specific proteins, promoting vascularization, modulating the interaction between epithelial and mesenchymal cells. Exosomes derived from DPSCs cultured under odontogenic differentiation conditions triggered dental pulp-like tissue regeneration in a tooth root-slice model, such as increased expression of DMP1, DPP, and active blood vessels [168]. Dental pulp is highly vascularized, and DPSC-exosomes contribute to the vascularization by activating the Notch signaling pathway, promoting pro-angiogenic factor expression, and tube formation of human umbilical vein endothelial cells [168].

DPSCs exosomes have been studied in a limited number of papers [167,168,169,170,171,172,173,174]. They showed a proangiogenic action [169,171], they can stimulate BMSCs migration and proliferation [175], and show strong immunomodulatory potential, affecting CD4+ T cells differentiation toward a T reg, despite a T helper 17 phenotype.

Exosomes derived from SCAP have been recently studied and promote BMMSCs-induced dentinogenesis with dentin-pulp complex regeneration, after subcutaneous implantation into SCID mice [176]. They showed anti-apoptotic activity on odontoblasts, also in inflammatory conditions, suggesting their protective role during inflammatory conditions [177].

Despite in vivo evidence and promising case reports, the translation of in situ tissue engineering into humans seems still unattained. Similarly, the regeneration of the dentin pulp complex as a whole owing to the interconnection of its components [178], albeit theoretically feasible [179], is hardly implementable at a clinical level to date.

## 4. Conclusions

The homeostasis of the oral cavity depends on the balance among the oral microbiome, the rate of inflammation, and the adaptive bone remodeling of the alveolar bone. Once inflammation takes over, irreversible conditions can occur, causing, for instance, periodontal disease and loss of alveolar bone. The anti-inflammatory and multi-differentiating abilities of MSCs allow their use in regenerative medicine. Adult MSCs can be harvested from different tissues and show a broad differentiation potential. Nonetheless, since MSCs maintain a memory of their origin, we believe that to treat diseases of the oral cavity, the usage of mouth-derived MSCs is advisable. These cells are present in different sites of the oral cavity and a relevant issue is also the possibility to treat patients with autologous MSCs, which further reduces the risk of adverse immune responses. Extracellular vesicles, which contain the curative potential of MSCs, are surely intriguing and open a wide variety of therapeutic perspectives. Future challenges are represented by the development of clinical protocols based on a standard preparation of biological products derived from each patient. To this end, the reduction of the cost/benefit ratio will play a major role in making the clinical procedures affordable. Similarly, accumulating clinical evidence on the safety and efficacy of the innovative protocols will support a paradigm shift from current to novel treatments. In most cases, therapy based on oral-cavity-derived MSCs seems to be quite efficient; however, due to relatively poor data available in literature, safety concerns may be raised, demanding that any potential risk be further investigated, above all in terms of long-lasting observations. One of the few and most recent works reporting results on MSCs’ safety investigated the pulp regenerative potential and transplantation safety of DPSCs in pulpectomized teeth in dogs, showing neither toxicity nor adverse events after their transplantation [180]. Based on these premises, furthermore, in vivo studies regarding potential adverse effects of these MSCs are mandatory.

## Figures and Tables

**Table 1:** Source and main activities of oral cavity-derived MSCs.

Name	Source	Regeneration Role
**DPSCs**	Dental Pulp	angiogenic potential [30], formation of dentin-pulp-like complex in empty root canal spaces [31,32,33], dentin repair [34], bone formation [39,40,41,42]
**SHED**	Exfoliated deciduous teeth	formation of dentin-like or pulp-like tissue [13], differentiation into endothelial cells [49,50], angiogenic ability [51,52], osteoinductive and osteogenic potential [53,54]
**PDLSCs**	Periodontal ligament	regeneration of PDL tissue [14,15,77], lower osteoinductive potential than DPSCs and SHED [56,57,58]
**SCAPs**	Apical papilla	remodeling and differentiation into dentin [15,16], root development and regeneration [62]
**DFSCs**	Dental follicle	odontogenic potential: dentin, root regeneration [67], and periodontal differentiation [17]
**GMSCs**	Gingiva	osteogenic potential in vitro [68,71], gingival lesion treatment [72,78], periodontal ligament regeneration in rats [48]
**BFPSCs**	Buccal fat pad	osteogenic potential [73,74]

## Data Availability

Not applicable.
